# X-linked dominant *RPGR* gene mutation in a familial Coats angiomatosis

**DOI:** 10.1186/s12886-020-01791-5

**Published:** 2021-01-14

**Authors:** Marcella Nebbioso, Federica Franzone, Alessandro Lambiase, Maurizio La Cava, Fabiana Mallone, Antonio Pizzuti, Enrica Marchionni

**Affiliations:** 1 Department of Sense Organs, Faculty of Medicine and Odontology, Sapienza University of Rome, p. le A. Moro 5, 00185 Rome, Italy; 2 Department of Sense Organs, Sapienza University of Rome, Viale del Policlinico 155, 00161 Rome, Italy; 3 Department of Experimental Medicine, Faculty of Medicine and Odontology, Sapienza University of Rome, p. le A. Moro 5, 00185 Rome, Italy

**Keywords:** Case report, Coats’-type retinitis pigmentosa, Coats vasculopathy, Hereditary disease, *RPGR*- XLRP- Coats’-like retinitis

## Abstract

**Background:**

Retinitis Pigmentosa (RP) is the most frequent retinal hereditary disease and every kind of transmission pattern has been described. The genetic etiology of RP is extremely heterogeneous and in the last few years the large application of Next Generation Sequencing (NGS) approaches improved the diagnostic yield, elucidating previously unexplained RP causes and new genotype-phenotype correlations. The objective of this study was to reevaluate a previously reported family affected by Coats’-type RP without genetic diagnosis and to describe the new genetic findings.

**Case presentation:**

Cohort, prospective, and single-center observational family case. Three individuals of a family, consisting of a mother and four sons, with a Coats phenotype were revaluated after 25 years of clinical follow-up using visual acuity tests, ophthalmoscopy, Goldmann visual field, electroretinography (ERG), and spectral domain-optical coherence tomography (SD-OCT). Specifically, a RP NGS panel was performed on one member of the family and segregation analysis was required for the other affected and unaffected members. NGS analysis disclosed a *RPGR (Retinitis Pigmentosa GTPase Regulator)* gene truncating variant segregating with the phenotype in all the three affected members. *RPGR* mutations are reported as causative of an X-linked RP.

**Conclusions:**

This is the first reported family with a Coats’-type RP associated to a *RPGR* mutation and segregating as a dominant X-linked disease, confirming the hypothesis of the genetic origin of this condition and expanding the phenotypic spectrum of diseases caused by *RPGR* gene mutations. The Authors suggest *RPGR* gene screening mutations in patients presenting this phenotype.

## Background

Retinitis pigmentosa (RP) represents a heterogeneous group of hereditary retinal disorders primarily characterized by a gradual loss of rods followed by cones damage, with a slow progression towards blindness. The peculiar photoreceptor dysfunction, presenting as bone spicule pigmentation, is typically associated with the attenuation of retinal vessels and a pallor of the optic nerve [1].

Coats’ disease is defined as an idiopathic retinal vascular disorder with retinal telangiectasia and intraretinal and/or subretinal exudation. At first, no retinal or vitreal traction is present. The disease is generally unilateral and usually affecting young males [2]. In the past some Authors used the terms “Coats disease” or “Coats response” referring to fundus changes observed in several forms of exudative retinopathies [2, 3]. Over time several definitions have been used to describe this condition as “Coats’ syndrome”, “retinal vasculopathy of the Coats’ type”, “Coats’-like detachment” and “Coats’-type RP” [3–8].

The latter description is the one preferred by many Authors as it explains a rare complication of RP, first described by Zamorani in 1956 [9]. He hypothesized that a pituitary dysfunction was causative of the association of these two pathologies, present in the same eye [9]. More in detail, Coats’-type RP refers to a peculiar form of Coats disease observed in some patients with advanced forms of RP. Therefore, these two diseases are not separate nosological entities but Coats’-type RP is a kind of RP [8]. To date, the disease’s incidence is not well known; Kajiwara [4] and Pruett [10] reported that almost 1% – 4% of cases of RP present Coats’disease, though this incidence may well be underestimated, as only RP cases complicated by exudative detachment are referred to study centers.

Patients affected by Coats’-type RP are usually of a higher average age than Coats patients, with a mean age of 26.6 years. Association with autosomal dominant [11, 12], autosomal recessive [13] and X-linked forms of RP (XLRP) have been described [14].

Coats’-type RP is characterized by vascular abnormalities as aneurismal dilatation and telangiectatic retinal veins, yellow extravascular lipid deposition, and retinal detachment [8, 10]. Clinically, Coats’-type changes in RP differ significantly from classic Coats’ disease, with regards to older age, absence of gender predisposition, and location in the inferior-temporal retina [8, 10, 12, 15]. Due to the relative rarity of both lesions, the precise nature of the relationship between Coats’-type changes and RP is still obscure. To date Coats’ disease is considered non-hereditary by international scientific literature.

The aim of our research was to describe the phenotype and genetic analysis of a mother and two sons affected by Coats’-type RP. The application of Next Generation Sequencing (NGS) analysis was used in the hypothesis of a possible genetic cause consistent with an X-linked dominant transmission.

## Case presentation

A three-generation family was revaluated with a multidisciplinary approach after 25 years of clinical follow-up. At first examination the probands were two brothers aged 28 (III:1, Fig. 1) and 26 (III:2, Fig. 1) affected by RP associated to Coats’- type changes. Their mother aged 51 (II:2, Fig. 1) presented the same phenotype, while among her four sons, two females were asymptomatic (III:3 and III:4, Fig. 1). An affected brother (III:2) and an asymptomatic sister (III:3) were fraternal twins. The maternal grandfather (I:1, Fig. 1) was reported as affected by early blindness, but had already died at the time of the study.

**Fig. 1:**
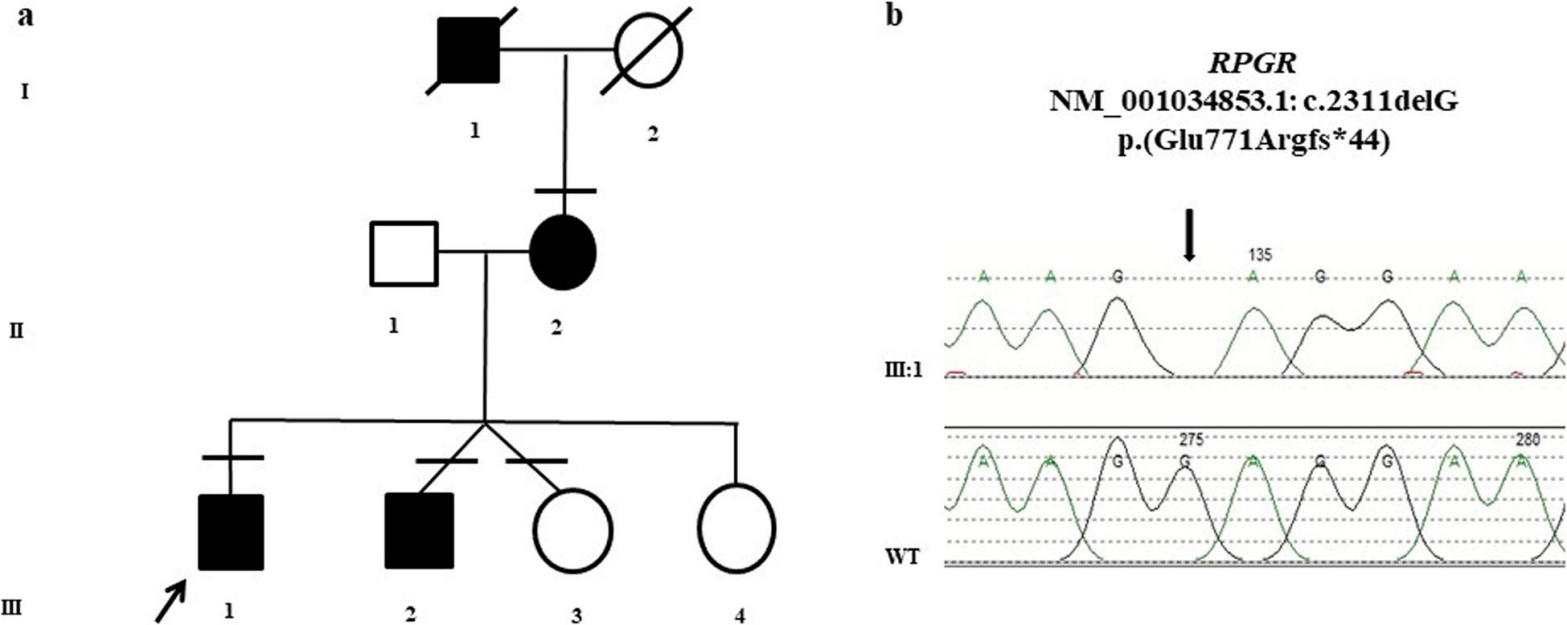
Pedigree of the family. **a** Black circle and squares indicate affected individuals. Black arrow indicates the proband who underwent NGS panel (III:1). Black lines at the top indicates available DNAs for segregation analysis (III:2, III:3 and II:2). **b** Sanger Sequencing Electropherograms showing on the top the proband (III:1) genotype of *RPGR *variant (black arrow) and below the wild-type (WT) genotype

Over the years, complete ophthalmological examination were performed, including the measurement of visual acuity, slit-lamp biomicroscopy, fundus examination, Goldmann visual field (GVF), visual evoked potentials (VEP), full-field electroretinography (ERG), multifocal ERG (optoelectronic stimulator Vision Monitor MonPak 120 by Metrovision, Pérenchies, France), spectral domain-optical coherence tomography (SD-OCT), and fluorescein angiography (FA) (Spectralis® HRA/OCT Heidelberg Engineering, Heidelberg Germany).

At first examination best corrected visual acuity (BCVA), tested with Snellen letters at a distance of 5 meters in II:2 patient (mother), was 20/400 and 20/200 in right and left eye, respectively. BCVA in III:1 patient (brother), was 20/25 and 20/200 in right and left eye, respectively. The left eye was amblyopic as a consequence of convergent strabismus diagnosed at the age of 9 years. BCVA in III:2 patient (brother) was 20/25 in both eyes. The two sisters (III:3 and III:4) showed normal fundus and electrophysiological examination.

**Fig. 2:**
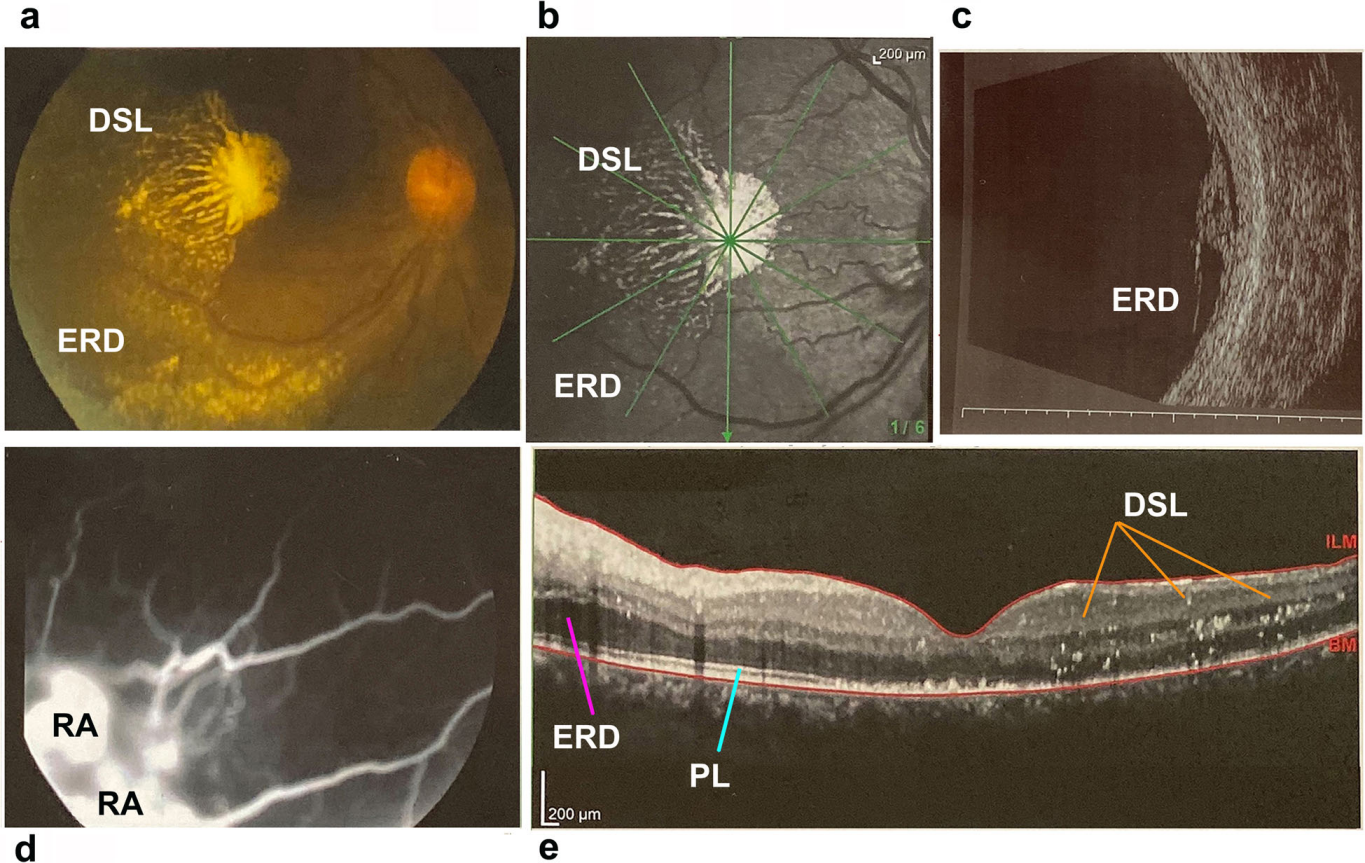
Images of the patient II:1 (mother). **a/b** The retinography and infrared photo show retinal exudations found from the center to the peripheral zones up to 2 papillary diameters and peripheral exudative retinal detachment (ERD) in the inferior and temporal sectors of right eye. **c/d** The retinal ultrasound and fluorescein angiography (FA) highlight exudative retinal detachment (ERD) area adjacent to retinal angiomatosis (RA) in the inferior and temporal sectors; hypo/anechogenic and hyperfluorescent areas, respectively. **e** The spectral domain-optical coherence tomography (SD-OCT) examination shows deposits of star-like hyper-reflective exudates (DSL) mainly present in the macular region. The photoreceptor layer (PL) is visible only in a portion of the macula in the patient affected from retinitis pigmentosa (RP)

**Fig. 3:**
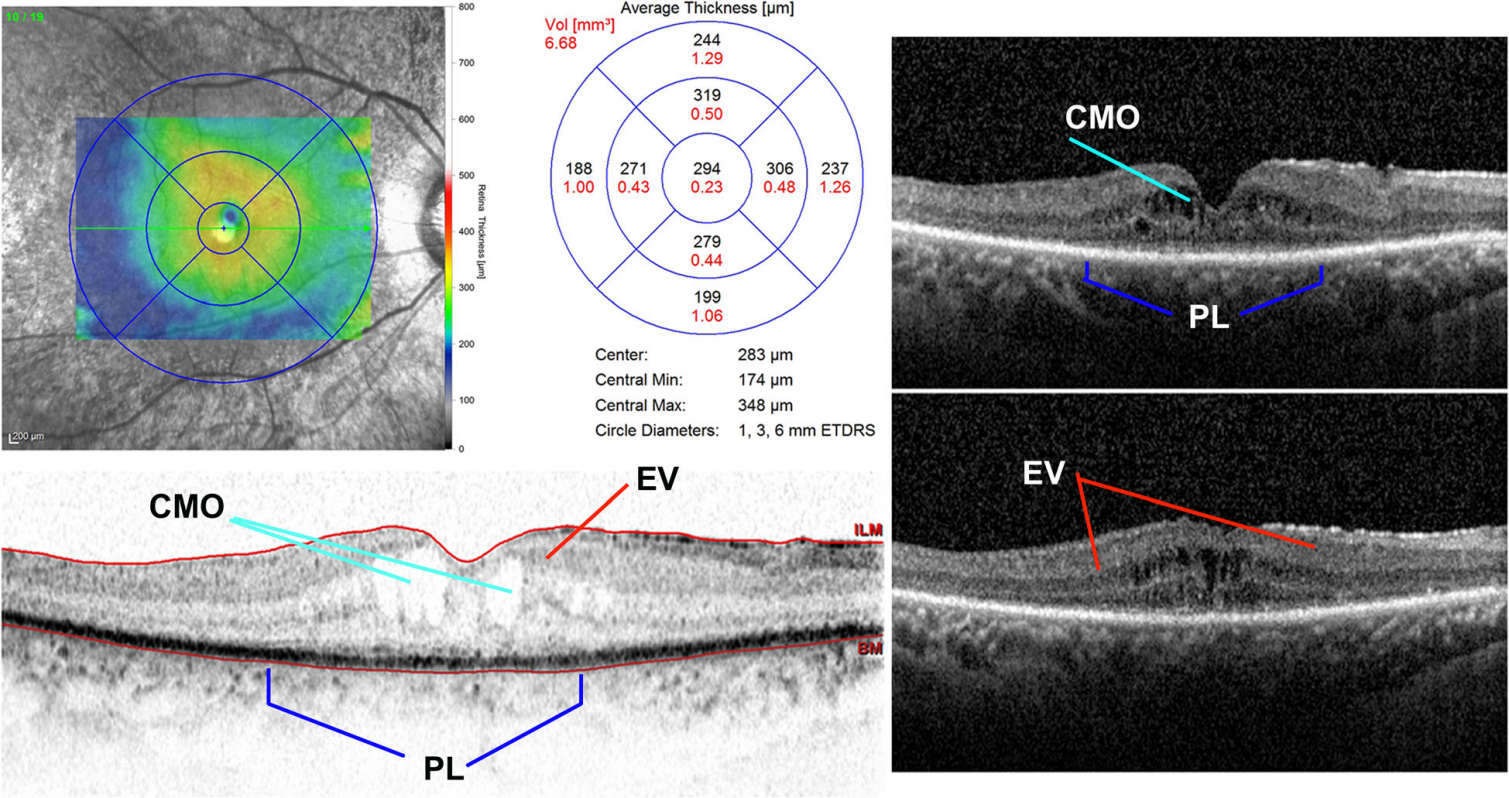
Spectral domain-optical coherence tomography (SD-OCT) of the III:1 patient (brother). Cystoid macular oedema (CMO) with areas of exudative vasculopathy (EV) for spongy appearance of the macular region in right eye. The photoreceptor layer (PL) is visible only in fovea of the patient affected from retinitis pigmentosa (RP)

In all three patients fundoscopic examination demonstrated typical retinal signs of RP associated to telangiectasias found in the peripheral zones up to 2 papillary diameters with variable expression in the three cases, while a peripheral exudative retinal detachment was observed in the inferior-temporal quadrants of their right eye (Fig. 2). In III:1 patient (brother) the treatment of the more severe cystoid macular oedema (CMO) with oral deflazacort (30 mg/day for 7 days, followed by gradual tapering of the dosage) resulted in slight reduction of the exudative vasculopathy (Fig. 3).

ERG, VEP, FA, and kinetic GVF demonstrated changes in keeping with the diagnosis of RP. Standard ERG elicited a retinal response of less than 10 µV (III:2 patient) or was undetectable (II:2 and III:1 patients). The patients (III:1 and III:2) had a RP with Coats’-type changes and the mother (II:2) had a more severe form of the same disease complicated by macular oedema, vitreitis (+ 3 cells) and epiretinal membranes. Unfortunately, the corticosteroid therapy administered to the mother resulted in a temporary benefit as regards visual acuity improvement.

In subsequent years, the 3 patients underwent surgery of cataract with implant of intraocular lens in both eyes, laser photocoagulation and cryotherapy in right eye. Afterwards, the slit-lamp examination revealed a more or less significant bilateral posterior subcapsular cataract.

Due to the progression of this complex disease, the patients have lost much of their visual capacity both in the binocular visual field, reduced at few percentage degrees, and in central visual acuity from 20/40 (III:2, younger brother) to 20/400 (II:2, mother) Snellen.

Previous genetic analysis failed to reveal mutations in rhodopsin (*RHO*, MIM # 180,380), peripherin (*PRPH2*, MIM # 179,605) and *RP2* genes (MIM # 300,757). More recently, patients have been revaluated and further genetic test have been performed. A RP gene panel was performed through NGS on genomic DNA extracted from saliva in one of the two affected brothers (III:1) and segregation analysis was performed on the other affected brother (III:2), his twin unaffected sister (III:3) and the mother (II:2).

Enzymatic fragmentation using Nextera Transposome System and hybridization-based enrichment of target regions using Illumina Nextera Rapid Capture Enrichment kit was performed on 50 ng of extracted DNA. Sequencing was performed on Illumina Miseq with paired-end protocol and generating 150 bp length reads. In this case the panel was expected to screen approximately 98.3% of the total panel regions with a minimum coverage of 25X. The panel included 70 genes, associated with RP and related retinal diseases. The panel detection rate (sensitivity) is > 99% with a minimum coverage of 10X, and the analytical specificity after direct sequencing is 99.99%. The identified variant was confirmed by Sanger sequencing performed with BigDye Terminator Sequencing Kit (v3.1), and a 3130xl Genetic Analyzer capillary sequencer (Fig. 1b).

In accordance with the Helsinki Declaration and Good Clinical Practice guidelines, the patients were informed about the use of their data and signed an informed consent. The study protocol was retrospectively registered.

The results of the molecular genetic analysis highlighted the following. NGS analysis disclosed in III:1 a hemizygous frameshift variant (NM_001034853.1: c.2311delG; NP_001030025.1:p.Glu771Argfs*44) in *RPGR* gene (Retinitis Pigmentosa GTPase Regulator, MIM # 312,610). Mutations in this gene are associated with one of the most frequent forms of XLRP (RP3, MIM # 300,029), X-linked Cone-Rod Dystrophy 1(CORDX1 MIM # 304,020), X-linked atrophic macular degeneration (MIM # 300,834), and a syndromic form of RP with sino-respiratory infections and with or without deafness (MIM # 300,455).

The same variant was consequently screened and identified in the affected hemizygous brother (III:2) and heterozygous mother (II:2), but not in the unaffected sister (III:3), thus confirming the phenotype segregation.

The variant is not annotated in GnomAD, one of the major population database (Genome Aggregation Database, v.2.1.1), it is reported in HGMD (The Human Gene Mutation Database) as Disease Mutation [16], and it can be classified as “Pathogenic” following the American College of Medical Genetics and Genomics criteria (PVS1, very strong; PM2, moderate; PP1, supporting criteria) [17].

It was previously reported in XLRP, without any clinical information [18].

The *RPGR* gene encodes multiple alternatively spliced isoforms but the precise number expressed in the human retina is still unclear. The two most important isoforms mutated in RP patients are: RPGR^1–19^ (amino acids 815; exons 1–19) and RPGR^ORF15^ (amino acids 1152; exons 1–15 and part of intron 15). Both isoforms share exons 1–15 and the N-terminus part. RPGR^ORF15^ has a different C-terminal domain, as exon 15 encodes for a protein domain rich in glutamic acids and glycines (Glu-Gly domain, EG domain) [19].

*RPGR* (NM_001034853.1:c.2311delG; p.Glu771Argfs*44) variant affects the RPGR^ORF15^ isoform. The variant is a G nucleotide deletion causing a frameshift and creating a premature stop codon 44 codons downstream, in alternative terminal exon 15, predicted to result in a truncated protein without the typical configuration of the normal C-terminal domain.

In human, out of frame deletions, duplications, or insertions are frequently found in exon ORF15 which is considered a mutational hotspot [19]. It is estimated that more than a half of *RPGR* mutations (~ 60–70%) are located in the ORF15 region and C-terminal truncations in the RPGR-ORF15 protein are well known causes of RPGR-associated phenotypes.

In this family, the *RPGR* variant segregates with Coats’-type RP as a dominant X-linked disease.

## Discussion and conclusions

This is the first reported family with a single gene mutation segregating with Coats’-type RP, in three patients. The data confirm the genetic origin of the pathology and expand the phenotypic spectrum of diseases caused by *RPGR* gene mutations. A few families have been reported with autosomal dominant [11, 12], autosomal recessive [13] and X-linked transmission [14], however the responsible gene(s) has/have not been identified. Actually, Demirci et al. reported a patient carrying a nonsense mutation in *RPGR* gene consistent with an X-linked recessive inheritance [20]. However, the other affected members of the family presented only RP without Coats’ phenotype and the Authors suggested a coincidental involvement of other (not identified) genetic and/or environmental factors independent of the RP-causing gene mutation [20]. The family we studied, shows that the Coats’-type changes are tightly linked to mutations in the same gene causative of the RP features. Therefore, after X-linked Cone-Rod Dystrophy 1(CORDX1 MIM # 304,020), X-linked recessive atrophic macular degeneration (MIM # 300,834), X-linked RP3 (MIM # 300,029) and X-linked RP with sino-respiratory infections and with or without deafness (MIM # 300,455), the new phenotype of RP with Coats’ changes could be added to the phenotypes caused by *RPGR* gene mutations.

In the family, the mother (II:2) and her two male sons (III:1; III:2) present the same RP phenotype with Coats’ changes, suggesting a dominant effect of the X-linked segregating mutation, in accordance to other reports showing *RPGR* heterozygous females with severe RP clinical symptoms, mimicking an autosomal dominant inheritance [21–23].

In 2002 Rozet et al. reported nine families affected by dominant X-linked RP caused by truncating mutations in exon ORF15 of the *RPGR* gene. In these families the phenotype was severe both in heterozygous females and hemizygous males [24] and no preferential X-inactivation was observed [25]. Later Andreasson et al. reported that RP «carriers» of *RPGR* ORF15 mutations showed advanced degrees of retinal degeneration, with significant difference between the two eyes and they attributed the phenotypic asymmetry to random X-inactivation without a formal confirmation [26]. Of note, the reported *RPGR* ORF15 mutations were truncating variants too.

Some Authors described a very large family affected by an X-linked dominant form of RP with a *RPGR* ORF15 frameshift variant predicted to result in premature truncation of the RPGR protein [21, 22]. They hypothesized the presence of modifier alleles and a possible dominant gain-of-function mechanism of some specific mutations. According to the Authors, families with dominant-acting *RPGR* ORF15 mutations may be mistaken as having an autosomal dominant inheritance, resulting in an incorrect genetic counseling [22]. Churchill et al. demonstrated that 7.8% of cases with mutations in *RPGR* gene were found in families whose initial diagnosis was autosomal dominant RP [27].

More recently, severe manifestations of X-linked RP were unexpectedly associated with truncating mutations in *RPGR* ORF15 region in one sporadic female carrier and in one patient with a family history compatible with autosomal dominant inheritance [28].

Some truncating mutations of *RPGR* ORF15 region seem to correlate with disease severity in female heterozygous carriers, although the underlying molecular mechanism has not been elucidated yet.

In literature few missense mutations located in other exons than ORF15 region of *RPGR* gene and presenting with X-linked dominant or semi-dominant transmission are reported [29, 30].

In the family we describe (Fig. 1), the presence of the same symptoms both in hemizygous and heterozygous affected patients adds further evidence of a dominant X-linked transmission associated with a *RPGR* ORF15 truncating mutation and for the first time with Coats’-type changes.

RP is characterized by high genetic and phenotypic heterogeneity, with more than 70 disease causing RP genes reported so far [31]. The introduction of targeted NGS panels enabled the parallel sequencing of many RP genes (and additional genes causing related disorders, if needed), improving the detection rate of disease-causing mutations in RP patients [28, 32]. This approach helps in clarifying the differential diagnosis and the detection of known or new disease-causing mutations is useful to disclose possible new genotype–phenotype correlations [28, 33]. The identification of additional phenotypes in such scenario, is fundamental to increase knowledge about pathways and biological processes involved in these diseases and to understand their pathogenesis. This valuable diagnostic tool, together with an accurate clinical and instrumental characterization of patients, has also direct impact on planning targeted therapies and follow-up in the era of novel upcoming therapeutic options (such as gene therapy) [32, 33].

The relationship between RP and Coats disease and the origin of the phenotype have been elusive for long times and in the last decades Coats-type changes have been ascribed to different causes. Particularly, in 1974 Witschel characterized histo-pathological findings of Coats’-type RP considering the disease as a primary vasculopathy, only expressed in the presence of RP as a vasodilatatory response to toxic products of photoreceptor/RPE degeneration [34]. Interestingly, in this report the retina and the vitreous were described as completely detached and collapsed with the presence of pigmented macrophages and hyalocytes. Surprisingly, optic nerve and ganglion cells were relatively preserved, while internal limiting membrane appeared thickened. Areas of fibrosis were observed in the peripheral retina associated with wide and thick pigmented cells aggregations [34]. The vessels of the posterior pole appeared occluded and sclerotic [8].

Solomon et al. instead attributed the pathology to vascular endothelium’s damage and disruption of the blood-retinal barrier, possibly driven by an autoimmune mechanism triggered by the degenerating photoreceptors [35].

Similarly to Coats disease, Lanier et al. suggested a primary endothelial cell dysfunction characterized by a vascular endothelium breakdown as the main cause of the typical Coats’-type RP exudative findings [13].

In addition, given the differences in gender and age of patients with Coats’-type RP compared to those with Coats disease, Banks Anderson postulated a different etiopathogenesis for the vasculopathy seen in Coats’-type RP. In view of these observations, some irritating substances released by the degenerating retina were considered toxic agents able to sustain a consequent immunological response with vascular abnormalities and damage [36]. According to Pruett, serous detachment, often described in these patients, is caused by chronic microvascular leakage. The inferior localization could be ascribed to gravitational pooling of the retinal fluid. As a consequence the retinal detachment would determine an inferior retinal oxygen deficit probably responsible of both telangiectatic and neovascularization changes [10].

According to literature, the patients affected by Coats’-type RP show a wide range of manifestations as visual impairments from moderate to severe, typical of RP, towards blindness [3–7]. It may also happen to accidentally observe patients with this pathology who are asymptomatic because the detachment generally occurs peripherally in areas that have already been damaged by RP. It is interesting to note that visual loss in these patients may be due to other conditions associated with the underlying disease such as neovascular glaucoma, vitreous hemorrhage and exudative detachment involving the posterior pole [3–7, 12, 13, 34–37]. Also, inflammatory signs that may be observed in RP patients with Coats’-type changes include anterior uveitis, vitreitis, optic disc oedema and vessel sheathing. Some forms of RP may demonstrate preretinal gliosis or cellophane maculopathy, and CMO, as well. Furthermore, although lipid deposition in Coats’-type RP has not been associated with elevated serum cholesterol, patient III:2 and the mother (II:2) underwent to an early cholecystectomy for stones. This is an interesting finding considering that the adult type of classic Coats disease is invariably associated with hypercholesterolemia [15] and hyperlipidaemia is common in RP patients [8]. Considering that many patients with XLRP have lower than normal blood levels of the long-chain polyunsaturated 3 fatty acid docosahexaenoic acid (DHA) [38], it can be speculated that changes in the bile acid pattern associated to the DHA deficiency might trigger gallstone formation in RP patients.

In this view, it is interesting to note that DHA signaling pathway has recently been demonstrated essential in photoreceptors preconditioning protection, as DHA and its derivatives regulate neuroprotective, anti-inflammatory, and anti-angiogenic bioactivity in photoreceptors and retinal pigment epithelial cells [39]. *RPGR* encodes the retinitis pigmentosa GTPase regulator protein, which is located in the connecting cilium, essential for the active transport of proteins between the inner and outer segments of photoreceptors. It has been hypothesized that loss of RPGR results in subtle defects that accumulate insult over time in the photoreceptors, leading to their dysfunction and degeneration [40]. Future investigations into the *RPGR* function and Docosanoid signaling pathway, could provide novel clues to elucidate the Coats’-type RP underlying mechanism.

Unfortunately, in our study we were unable to fully check the RPGR variant in other family members. The limitations of this study include the small number of patients studied which restrict the power to detect differences between subgroups.

In our study, systemic and topical steroids have been used with variable results in treatment of Coats-type RP [8]. In the present cases they proved to be successful temporarily, with improvement of the visual acuity in the patients affected from inflammatory signs as uveitis, vitreitis and CMO. In subsequent years, the 3 patients underwent surgery of cataract, laser photocoagulation and cryotherapy in right eye. Nevertheless, due to the progression of this complex disease, the patients have lost much of their visual capacity.

More cases will be necessary to speculate the genotype-phenotype correlations and we suggest to screen mutations on this gene in patients presenting Coats’-type RP to better elucidate the genetic origin and phenotypic expression of this condition both in males and females.

## Data Availability

The datasets produced and analyzed during the study are available from the corresponding author.

## Abbreviations

BCVA: Best corrected visual acuity
CMO: Cystoid macular oedema
DHA: Docosahexaenoic acid
DSL: Deposits of star-like hyper-reflective exudates
ERD: Exudative retinal detachment
ERG: Electroretinography
EV: Exudative vasculopathy
FA: Fluorescein angiography
GVF: Goldmann visual field
NGS: Next Generation Sequencing
PL: Photoreceptor layer
RA: Retinal angiomatosis
RP: Retinitis pigmentosa
RPGR gene: Retinitis Pigmentosa GTPase Regulator, MIM # 312610
SD-OCT: Spectral domain-optical coherence tomography
VEP: Visual evoked potentials

## References

[1] Verbakel SK, van Huet RAC, Boon CJF, den Hollander AI, Collin RWJ, Klaver CCW (2018). Non-syndromic retinitis pigmentosa. Prog Retin Eye Res..

[2] Sen M, Shields CL, Honavar SG, Shields JA (2019). Coats disease: an overview of classification, management and outcomes. Indian J Ophthalmol.

[3] Shields JA, Shields CL, Honavar SG, Demirci H (2001). Clinical variations and complications of coats disease in 150 cases: the 2000 Sanford Gifford Memorial Lecture. Am J Ophthalmol.

[4] Kajiwara Y (1980). Ocular complications of retinitis pigmentosa. Association with Coats’ syndrome. Jpn J Clin Ophthalmol.

[5] Schmidt D, Faulborn J (1972). Familiares Vorkommen Coats-syndrom kombiniert mit Retinpathia pigmentosa. Klin Monatsbl Augenheilkd.

[6] Fogle JA, Welch RB, Green WR (1978). Retinitis pigmentosa and exudative vasculopathy. Arch Ophthalmol.

[7] Ide CH, Khan JA, Podolsky TL, Strickland MP, Wilson RJ (1987). Coat’s type retinitis pigmentosa. Klin Monbl Augenheilkd.

[8] Khan JA, Ide CH, Strickland MP (1988). Coats’-type retinitis pigmentosa. Surv Ophthalmol.

[9] Zamorani G (1956). Una rara associazione di retinite di Coats con retinite pigmentosa. Gior Ital Oftalmol.

[10] Pruett RC (1983). Retinitis pigmentosa: clinical observations and correlations. Trans Am Ophthalmol Soc.

[11] Perrier M, Gauthier D, Sébag M, Marcil G (2004). Coats’-type retinitis pigmentosa: first reported case of presumed vertical transmission. Can J Ophthalmol.

[12] Spallone A, Carlevaro G, Ridling P (1985). Autosomal dominant retinitis pigmentosa and Coats’-like disease. Int Ophthalmol.

[13] Lanier JD, McCrary JA, Justice J (1976). Autosomal recessive retinitis pigmentosa and Coats disease: a presumed familial incidence. Arch Ophthalmol.

[14] Bansal S, Saha N, Woon WH (2011). The management of “Coats’ response” in a patient with x-linked retinitis pigmentosa-a case report. ISRN Surg.

[15] Jiang Y, Lim J, Janowicz M (2017). Cholesterol crystals secondary to coats-like response with retinitis pigmentosa. JAMA Ophthalmol.

[16] Stenson PD, Ball EV, Mort M, Phillips AD, Shiel JA, Thomas NS (2003). Human Gene Mutation Database (HGMD): 2003 update. Hum Mutat.

[17] Richards S, Aziz N, Bale S, Bick D, Das S, Gastier-Foster J (2015). Standards and guidelines for the interpretation of sequence variants: a joint consensus recommendation of the American College of Medical Genetics and Genomics and the Association for Molecular Pathology. Genet Med.

[18] Wang DY, Chan WM, Tam PO, Baum L, Lam DS, Chong KK (2005). Gene mutations in retinitis pigmentosa and their clinical implications. Clin Chim Acta.

[19] Vervoort R, Lennon A, Bird AC, Tulloch B, Axton R, Miano MG (2000). Mutational hot spot within a new RPGR exon in X-linked retinitis pigmentosa. Nat Genet.

[20] Demirci FY, Rigatti BW, Mah TS, Gorin MB (2006). A novel RPGR exon ORF15 mutation in a family with X-linked retinitis pigmentosa and Coats’-like exudative vasculopathy. Am J Ophthalmol.

[21] Mears AJ, Hiriyanna S, Vervoort R, Yashar B, Gieser L, Fahrner S (2000). Remapping of the RP15 locus for X-linked cone-rod degeneration to Xp11.4-p21.1, and identification of a de novo insertion in the RPGR exon ORF15. Am J Hum Genet.

[22] Wu DM, Khanna H, Atmaca-Sonmez P, Sieving PA, Branham K, Othman M (2010). Long-term follow-up of a family with dominant X-linked retinitis pigmentosa. Eye..

[23] Hasan SM, Azmeh A, Mostafa O, Megarbane A (2016). Coat’s like vasculopathy in leber congenital amaurosis secondary to homozygous mutations in CRB1: a case report and discussion of the management options. BMC Res Notes.

[24] Rozet JM, Perrault I, Gigarel N, Souied E, Ghazi I, Gerber S (2002). Dominant X linked retinitis pigmentosa is frequently accounted for by truncating mutations in exon ORF15 of the RPGR gene. J Med Genet.

[25] Souied E, Segues B, Ghazi I, Rozet JM, Chatelin S, Gerber S (1997). Severe manifestations in carrier females in X linked retinitis pigmentosa. J Med Genet.

[26] Andréasson S, Breuer DK, Eksandh L, Ponjavic V, Frennesson C, Hiriyanna S (2003). Clinical studies of X-linked retinitis pigmentosa in three Swedish families with newly identified mutations in the RP2 and RPGR-ORF15 genes. Ophthalmic Genet.

[27] Churchill JD, Bowne SJ, Sullivan LS, Lewis RA, Wheaton DK, Birch DG (2013). Mutations in the X-linked retinitis pigmentosa genes RPGR and RP2 found in 8.5% of families with a provisional diagnosis of autosomal dominant retinitis pigmentosa. Invest Ophthalmol Vis Sci.

[28] Birtel J, Gliem M, Mangold E, Müller PL, Holz FG, Neuhaus C (2018). Next-generation sequencing identifies unexpected genotype-phenotype correlations in patients with retinitis pigmentosa. PLoS One..

[29] Al-Maskari A, O’Grady A, Pal B, McKibbin M (2009). Phenotypic progression in X-linked retinitis pigmentosa secondary to a novel mutation in the RPGR gene. Eye.

[30] Banin E, Mizrahi-Meissonnier L, Neis R, Silverstein S, Magyar I, Abeliovich D (2007). A nonancestral RPGR missense mutation in families with either recessive or semi-dominant X-linked retinitis pigmentosa. Am J Med Genet A.

[31] RetNet. https://sph.uth.edu/retnet/, last accessed 23 Nov 2020.

[32] Jespersgaard C, Fang M, Bertelsen M, Dang X, Jensen H, Chen Y (2019). Molecular genetic analysis using targeted NGS analysis of 677 individuals with retinal dystrophy. Sci Rep.

[33] Strafella C, Caputo V, Pagliaroli G, Iozzo N, Campoli G, Carboni S (2019). NGS Analysis for molecular diagnosis of retinitis pigmentosa (RP): detection of a novel variant in PRPH2 gene. Genes.

[34] Witschel H (1974). Retinopathia pigmentosa and “Morbus Coats”. Klin Monbl Augenheilkd.

[35] Solomon A, Banin E, Anteby I, Benezra D (1999). Retinitis pigmentosa, Coats disease and uveitis. Eur J Ophthalmol.

[36] Banks Anderson W, Wadsworth JA, Landers MB (1977). Retinitis pigmentosa and a retinal vasculopathy of the Coats type. Adv Exp Med Biol.

[37] Tortorella P, D’Ambrosio E, Iannetti L, De Marco F, La Cava M (2015). Correlation between visual acuity, inner segment/outer segment junction, and cone outer segment tips line integrity in uveitic macular edema. Biomed Res Int.

[38] Hoffman DR, Locke KG, Wheaton DH, Fish GE, Spencer R, Birch DG (2004). A randomized placebo-controlled clinical trial of docosahexaenoic acid supplementation for X-linked retinis pigmentosa. Am J Ophthalmol.

[39] Knott EJ, Gordon WC, Jun B, Do K, Bazan NG (2018). Retinal pigment epithelium and photoreceptor preconditioning protection requires docosanoid signaling. Cell Mol Neurobiol.

[40] Khanna H (2018). More than meets the eye: current understanding of RPGR function. Adv Exp Med Biol.

